# A multilocus timescale for oomycete evolution estimated under three distinct molecular clock models

**DOI:** 10.1186/1471-2148-14-101

**Published:** 2014-05-12

**Authors:** Nahill H Matari, Jaime E Blair

**Affiliations:** 1 Department of Biology, Franklin & Marshall College, Lancaster, PA, USA

**Keywords:** Oomycetes, Divergence times, Bayesian inference, Molecular clock, Gene expression regulation

## Abstract

**Background:**

Molecular clock methodologies allow for the estimation of divergence times across a variety of organisms; this can be particularly useful for groups lacking robust fossil histories, such as microbial eukaryotes with few distinguishing morphological traits. Here we have used a Bayesian molecular clock method under three distinct clock models to estimate divergence times within oomycetes, a group of fungal-like eukaryotes that are ubiquitous in the environment and include a number of devastating pathogenic species. The earliest fossil evidence for oomycetes comes from the Lower Devonian (~400 Ma), however the taxonomic affinities of these fossils are unclear.

**Results:**

Complete genome sequences were used to identify orthologous proteins among oomycetes, diatoms, and a brown alga, with a focus on conserved regulators of gene expression such as DNA and histone modifiers and transcription factors. Our molecular clock estimates place the origin of oomycetes by at least the mid-Paleozoic (~430-400 Ma), with the divergence between two major lineages, the peronosporaleans and saprolegnialeans, in the early Mesozoic (~225-190 Ma). Divergence times estimated under the three clock models were similar, although only the strict and random local clock models produced reliable estimates for most parameters.

**Conclusions:**

Our molecular timescale suggests that modern pathogenic oomycetes diverged well after the origin of their respective hosts, indicating that environmental conditions or perhaps horizontal gene transfer events, rather than host availability, may have driven lineage diversification. Our findings also suggest that the last common ancestor of oomycetes possessed a full complement of eukaryotic regulatory proteins, including those involved in histone modification, RNA interference, and tRNA and rRNA methylation; interestingly no match to canonical DNA methyltransferases could be identified in the oomycete genomes studied here.

## Background

Eukaryotic diversity is primarily microbial, with multicellularity restricted to a few distinct lineages (plants, animals, fungi, and some algae). While the Proterozoic fossil record contains an abundance of organic-walled, often ornamented, microfossils interpreted as eukaryotes, evidence for the origins and diversification of specific lineages of microbial eukaryotes is rare, especially for those groups with few diagnostic morphological characters [1]. Molecular clock methods therefore provide the only avenue for elucidating the evolutionary history of some lineages. With the recognition that a single rate (“strict”) molecular clock as originally proposed by Zuckerkandl and Pauling [2,3] was often inadequate in light of rate variation among organisms, early studies suggested the use of local clocks or the removal of lineages that violated the assumption of rate homogeneity (reviewed in [4]). The continued development of molecular clock methodologies over the past two decades has allowed for the estimation of divergence times under more complex models of rate variation. Initial “relaxed clock” methods, such as non-parametric rate smoothing [5] and penalized likelihood [6], allowed rates to vary but sought to minimize large differences between parent and descendent branches. Additionally, Bayesian relaxed clock methods allow rates to vary among lineages but assume autocorrelation by drawing the rate of a descendent branch from a distribution whose mean is determined by the rate of the parent branch [7,8]; other Bayesian methods relax this assumption of autocorrelation for the co-estimation of phylogeny and divergence times [9]. Most recently, a random local clock model approach has been proposed which allows rate changes to occur along any branch in a phylogeny; this method allows users to directly test various local clock scenarios against a strict clock model of no rate changes [10].

In addition to improved modeling of rate variation, newer molecular clock methods are also able to better incorporate calibration uncertainty into the estimation of divergence times. Early methods treated fossil calibrations as fixed points (from which rates were derived); newer methods utilize probability distributions to better reflect the paleontological uncertainty of a fossil’s phylogenetic position in relation to modern organisms [11,12], as well as variance around the numerical age of geologic formations. However, some authors have already shown that modeling fossil probability distributions under different assumptions can have significant impacts on divergence time estimation [13], illustrating that rate calibration is still an important source of potential error in molecular clock studies.

In this study we have focused on the fungal-like oomycetes (Peronosporomycetes *sensu*[14]), a group of heterotrophic eukaryotes closely related to diatoms, brown algae, and other stramenopiles [15]. A close relationship among stramenopiles, alveolates, and several photosynthetic eukaryotes with red algal-derived plastids was previously suggested as the supergroup Chromalveolata [16]. However molecular studies have supported a grouping of stramenopiles and alveolates with the non-photosynthetic rhizarians (“SAR” *sensu*[17]), excluding other photosynthetic lineages; the recently revised eukaryote classification has now formalized the Sar supergroup [18]. Many oomycetes are saprotrophic in aquatic and terrestrial ecosystems, however several devastating pathogens are known, such as *Phytophthora infestans*, the causal agent of late blight in solanaceous plant hosts [19]. Some orders are primarily pathogenic, such as the Peronosporales and Albuginales, while others are composed of both pathogenic and saprotrophic members, such as the Pythiales, Saprolegniales, Leptomitales, and Rhipidiales [20]. Several basal lineages, such as the Eurychasmales and Haliphthorales, are known primarily as pathogens of marine algae and crustaceans, leading some to suggest that the oomycetes may be “hard-wired” for pathogenic lifestyles [15].

The earliest robust fossil evidence of oomycetes comes from the Lower Devonian (Pragian, ~408 Ma) Rhynie Chert [21]. Thick-walled, ornamented structures interpreted as oogonium-antheridium complexes [22], as well as thin-walled polyoosporous oogonia [23], are well preserved in association with degraded plant debris and cyanobacteria-dominated microbial mats. More recent oomycete fossils occur in the Carboniferous, where evidence for endophytic [24] and perhaps parasitic [25,26] interactions with plant hosts is more compelling. Additionally, the fossil species *Combresomyces cornifer* originally described from Lower Carboniferous chert in central France [27] has also been identified in Middle Triassic silicified peat from Antarctica [28], providing an intriguing example of geographic range and morphological stasis over roughly 90 million years of oomycete evolution [29].

This is the first study to estimate divergence times within the oomycetes using molecular clock methods. Previous studies have typically included a single representative within a larger study of eukaryotic evolution [30-32], or have used oomycetes to root the analysis [33,34]. As there is little *a priori* information on the tempo of evolution within oomycetes, here we estimate divergence times under three distinct molecular clock models: a single-rate strict clock, a relaxed clock with uncorrelated rates modeled under a lognormal distribution (UCLD), and a random local clock model. The availability of several complete genome sequences for oomycetes, diatoms, and a brown alga allowed us to carefully curate a dataset of 40 orthologs for divergence time estimation; we chose to focus on known regulators of eukaryotic gene expression to investigate their presence and level of conservation within pathogenic oomycetes. While the performance of the three models differed, the estimated divergence times suggested that oomycetes diverged from other stramenopiles by at least the mid-Paleozoic, and that two major lineages, the peronosporaleans and saprolegnialeans, diverged in the early Mesozoic, approximately 200 Ma after the first appearance of oomycetes in the fossil record.

## Results

### Regulators of gene expression in oomycetes

Complete genome sequences from eighteen species were examined (Table 1). A total of 70 genes involved in the regulation of gene expression were examined for homology in *Phytophthora infestans* (Table 2); homologs of two genes (Drosha-like; TFIIH, Ssl1 subunit) could not be identified in *P. infestans* but were present in other oomycetes. In general, oomycetes possess a full complement of canonical transcription factors and genes involved in chromatin modification, including multiple histone acetyltransferases, deacetylases, and methyltransferases (Table 2). Proteins known to be involved in post-transcriptional gene silencing [35] were identified in our search, including homologs of Argonaut, Dicer, RNA-dependent RNA polymerase, double-stranded RNA binding proteins, and an RNaseIII-domain containing protein (Table 2). A recent study has shown that these genes are expressed and functional in *P. infestans*[36]. However, unlike the previous study, we were able to identify a second Dicer-like homolog in the genomes of other oomycetes that is absent in *P. infestans*; these sequences showed more similarity to human and *Drosophila* Drosha proteins than to other Dicer homologs (data not shown). Two distinct groups of Argonaut proteins were identified in the oomycetes, as well as two types of double-stranded RNA binding proteins (Table 2). While no homologs to canonical eukaryotic DNA methyltransferases could be identified, a homolog of DNA methyltransferase 1-associated protein was present in all the genomes analyzed here. Several genes involved in RNA methylation were also found (Table 2).

**Table 1:** Species with complete genome sequences included in this study

**Species**	**Strain/version**	**Genome source**	**Reference**
*Achlya hypogyna*	ATCC48635	unpublished data	(unpublished)
*Albugo laibachii*	Nc14	NCBI BLAST (http://blast.ncbi.nlm.nih.gov)	[37]
*Ectocarpus siliculosus*	Ec32	BOGAS (http://bioinformatics.psb.ugent.be)	[38]
*Fragilariopsis cylindrus*	CCMP1102 v1.0	DOE-Joint Genome Institute (http://www.jgi.doe.gov)	(unpublished)
*Hyaloperonospora arabidopsis*	Emoy2 v8.3.2	Virginia Bioinformatics Institute (http://www.vbi.vt.edu)	[39]
*Phaeodactylum tricornutum*	CCAP1055/1 v2.0	DOE-Joint Genome Institute (http://www.jgi.doe.gov)	[40]
*Phytophthora capsici*	LT1534 v11.0	DOE-Joint Genome Institute (http://www.jgi.doe.gov)	[41]
*Phytophthora cinnamomi*	v1.0	DOE-Joint Genome Institute (http://www.jgi.doe.gov)	(unpublished)
*Phytophthora infestans*	T30-4	Broad Institute (http://www.broadinstitute.org)	[42]
*Phytophthora parasitica*	INRA-310	Broad Institute (http://www.broadinstitute.org)	(unpublished)
*Phytophthora ramorum*	Pr-102 v1.1	DOE-Joint Genome Institute (http://www.jgi.doe.gov)	[43]
*Phytophthora sojae*	P6497 v3.0	DOE-Joint Genome Institute (http://www.jgi.doe.gov)	[43]
*Pseudo-nitzschia multiseries*	CLN-47 v1.0	DOE-Joint Genome Institute (http://www.jgi.doe.gov)	(unpublished)
*Pythium ultimum*	BR144 v4.0	Pythium Genome Database (http://pythium.plantbiology.msu.edu)	[44]
*Saprolegnia parasitica*	CBS223.65	Broad Institute (http://www.broadinstitute.org)	[45]
*Tetrahymena thermophila*	SB210 v2008	Tetrahymena Genome Database (http://ciliate.org)	[46]
*Thalassiosira pseudonana*	CCMP1335 v3.0	DOE-Joint Genome Institute (http://www.jgi.doe.gov)	[47]
*Thraustotheca clavata*	ATCC34112	unpublished data	(unpublished)

**Table 2:** Conserved regulators of gene expression evaluated for divergence time analysis

**Gene**	**Domains**^**a**^	**Reference**^**b**^
*Chromatin modification*		
Anti-silencing factor Asf1	asf1	PITG_17091
Brahma-like	HAS, SNF2 N-terminal, Helicase conserved C-terminal, Bromodomain	PITG_19037
Chromodomain-containing protein (A)	2x Chromo, SNF2 N-terminal, Helicase conserved C-terminal	PITG_15837
Chromodomain-containing protein (B)	[PDZ, QLQ], 2x Chromo, SNF2 N-terminal, Helicase conserved C-terminal, PHD-finger, PHD-like (zf-HC5HC2H_2)	PITG_10083
Chromodomain-containing protein (C)	PHD-finger, 2x Chromo, SNF2 N-terminal, Helicase conserved C-terminal	PITG_00140
Chromodomain-containing protein (D)	[Chromo], Bromodomain, PHD, Chromo, [PDZ], SNF2 N-terminal, Helicase conserved C-terminal, PHD, [PHD], PHD-like (zf-HC5HC2H), PHD	PITG_03401
CXXC zinc finger containing protein	[SNF2 N-terminal], 2x CXXC zinc finger, [FHA]	PITG_03547
DNA methyltransferase 1-associated protein	[DNA methyltransferase 1-associated protein]	PITG_15785
ESA1-like histone acetyltransferase	Tudor-knot RNA binding, MOZ/SAS	PITG_01456
GCN5-like histone acetyltransferase	GNAT Acetyltransferase, Bromodomain	PITG_20197
HAT1-like histone acetyltransferase	HAT1 N-terminus, [GNAT Acetyltransferase]	PITG_00186
KAT11 domain histone acetyltransferase (A)	TAZ zinc finger, Bromodomain, [PHD], KAT11, ZZ zinc finger, TAZ zinc finger	PITG_07302
KAT11 domain histone acetyltransferase (B)	[TAZ, TAZ], Bromodomain, [DUF902], [PHD], KAT11, [ZZ, TAZ]	PITG_06533
KAT11 domain histone acetyltransferase (C)	Bromodomain, [PHD], KAT11	PITG_18027
KAT11 domain histone acetyltransferase (D)	Bromodomain, KAT11	PITG_08587
Histone deacetylase HDA1	histone deacetylase	PITG_01897
Histone deacetylase HDA2	[ankyrin repeats], histone deacetylase	PITG_08237
Histone deacetylase HDA4	histone deacetylase	PITG_05176
Histone deacetylase HDA5	histone deacetylase	PITG_15415
Histone deacetylase HDA6	histone deacetylase	PITG_21309
Histone deacetylase HDA7	histone deacetylase	PITG_12962
Histone deacetylase HDA8	histone deacetylase	PITG_01911
Histone deacetylase HDA9	histone deacetylase	PITG_04499
DOT1-like histone methyltransferase	DOT1	PITG_00145
Histone-lysine N-methyltransferase	Bromodomain, PHD-like zinc-binding (zf-HC5HC2H), F/Y-rich N-terminus, SET	PITG_20502, PITG_04185
Protein methyltransferase w/bicoid	Methyltransferase, bicoid-interacting protein 3	PITG_14915
SLIDE domain-containing protein (A)	DUF1898, SNF2 N-terminal, Helicase conserved C-terminal, SLIDE, [myb-like DNA-binding], HMG box	PITG_02286
SLIDE domain-containing protein (B)	SNF2 N-terminal, Helicase conserved C-terminal, [HAND], SLIDE	PITG_17273
SSRP1 subunit, FACT complex	Structure-specific recognition protein, Histone chaperone Rttp106-like	PITG_14260
*RNA Methylation*		
FtsJ-like rRNA Methyltransferase (A)	FtsJ-like methyltransferase	PITG_09405
FtsJ-like rRNA Methyltransferase (B)	FtsJ-like methyltransferase	PITG_06848
FtsJ-like rRNA Methyltransferase (C)	FtsJ-like methyltransferase	PITG_16337
Spb1-like rRNA Methyltransferase	FtsJ-like methyltransferase, DUF3381, Spb1 C-terminal domain	PITG_00663
Guanosine 2'O tRNA methyltransferase	CCCH zinc finger, U11-48 K CHHC zinc finger, TRM13 methyltransferase	PITG_04858
N2,N2-dimethylguanosine tRNA methyltransferase	N2,N2-dimethylguanosine tRNA methyltransferase (TRM)	PITG_10166
MnmA-like tRNA 2'-thiouridylase	tRNA methyltransferase	PITG_08823
*RNA Silencing*		
Argonaute (A)	DUF1785, PAZ, Piwi	PITG_04470, PITG_04471
Argonaute (B)	DUF1785, PAZ, Piwi	PITG_01400, PITG_01443, PITG_01444
Dicer-like	[DEAD/H box helicase], dsRNA binding, 2x Rnase III domains	PITG_09292
Drosha-like	2x Rnase III domains, [dsRNA binding]	Psojae_300435
dsRNA-binding protein	dsRNA binding	PITG_12183
dsRNA-binding protein w/ Bin3	[methyltransferase], dsRNA binding, Bicoid-interacting 3	PITG_03262
RnaseIII domain protein	Rnase III domain, [dsRNA binding]	PITG_08831
RNA-dependant RNA polymerase	DEAD/H box helicase, Helicase conserved C-terminal, RdRP domain, [NTP transferase]	PITG_10457
*Transcription factors*		
Histone-like CBF/NF-Y	CBF/NF-Y [CENP-S associated centromere protein X]	PITG_00914
Histone-like CBF/NF-Y w/HMG	HMG, CBF/NF-Y	PITG_19530
Med17 subunit of Mediator complex	Med17	PITG_03899
p15 transcriptional coactivator	2x PC4	PITG_07058
TFIIB	TFIIB zinc-binding, 2x TFIIB	PITG_14596
TFIID, TATA-binding protein (A)	2x TBP	PITG_07312
TFIID, TATA-binding protein (B)	2x TBP, [DUF3378]	PITG_12304
TFIID, TATA-binding protein (C)	TBP, [2x DUF3378], TBP	PITG_06201
TFIID, TAF1 subunit	DUF3591, Bromodomain	PITG_02547
TFIID, TAF2 subunit	Peptidase M1, [HEAT repeat]	PITG_18882, PITG_14044
TFIID, TAF5 subunit	TFIID 90 kDa, 5x WD domain	PITG_16023
TFIID, TAF6 subunit	TAF, DUF1546, [HEAT repeat]	PITG_03978
TFIID, TAF8 subunit	Bromodomain (histone-like fold), TAF8 C-terminal	PITG_18355
TFIID, TAF9 subunit	TFIID 31 kDa	PITG_04860
TFIID, TAF10 subunit	TFIID 23–30 kDa	PITG_07637, PITG_14668
TFIID, TAF12 subunit	TFIID 20 kDa	PITG_00683
TFIID, TAF14 subunit	YEATS	PITG_01229
TFIIE, alpha subunit	TFIIEalpha	PITG_08403
TFIIF, alpha subunit	TFIIFalpha	PITG_02327
TFIIF, beta subunit	TFIIFbeta	PITG_10081
TFIIH, Rad3 subunit	DEAD 2, DUF1227, Helicase C-terminal	PITG_15696
TFIIH, Ssl1 subunit	Ssl1-like, TFIIH c1-like	Psojae_345458
TFIIH, Tfb1 subunit	[TFIIH p62 N-terminal], BSD	PITG_03523
TFIIH, Tfb2 subunit	Tfb2	PITG_15486
TFIIH, Tfb4 subunit	Tfb4	PITG_00220
TFIIIB, Brf1-like subunit	TFIIB zinc-binding, 2x TFIIB, Brf1-like TBP-binding	PITG_16669

^a^Domains in brackets indicate missing or non-significant matches in some species.

^b^Reference sequences from *P. infestans* T30-4 (PITG) or *P. sojae* P6497 v3.0.

### Divergence time analyses

**Figure 1:**
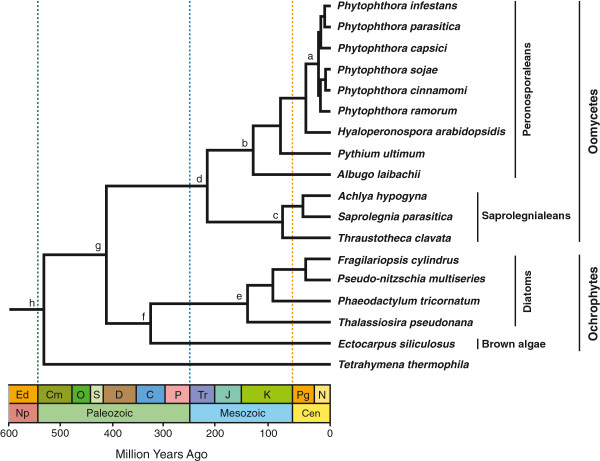
**Timetree of oomycete evolution.** Divergence times shown were estimated under the random local clock model. Vertical dashed lines indicate boundaries between geologic eras.

Robust orthology relationships could be determined for 52 out of the initial 70 datasets; 40 of these datasets contained minimal missing data and were used to estimate divergence times (see Additional file 1 for a list of genes included in the analysis). Calibration priors were modeled with a gamma distribution in order to assign higher probabilities to divergence times somewhat older than the hard bound (offset value); initial tests with lognormal priors produced very similar divergence times (data not shown). Five independent analyses of 50 million generations each were run under each of the three models, with the random local clock model being the most computationally intensive. Strict clock and UCLD analyses run on an iMac (10.8.5) desktop with a 2.7 GHz Intel core i5 processor took approximately seven days. Random local clock analyses run on a Linux (Mint14) desktop with a 3.3 GHz Xeon quad core processor took approximately 30 days. Posterior distributions on parameters were identical across all five runs under the strict clock model. Parameter distributions were consistent and overlapping for all five runs under the UCLD model with only one run deviating for the estimate of the root height (700 Ma versus approximately 500 Ma in the other four runs), however all runs showed weak evidence of convergence even after 50 million generations. One run under the random local clock model failed to converge; of the four successful runs, parameter distributions were consistent and overlapping with only one run deviating for the rate estimate (1.76 × 10^−3^ versus 1.88 × 10^−3^ for the other three runs). Log and tree files for two of the five runs with the highest effective sample size (ESS) for the likelihood parameter were then combined; under the strict clock model, all five runs performed equally, so the first two runs were combined. Analyses run without data (Prior Only) resulted in time estimates that were markedly different from those obtained with the full dataset for the majority of nodes (Table 3), suggesting that our divergence time estimates were driven by the data themselves and not by settings on the calibration priors. Divergence times among oomycete lineages were consistent among all three models (Table 3), however estimates under the UCLD model may have been influenced by poor mixing as several parameters showed ESS values less than 200 (Tables 3 and 4). The resulting timetree suggests an origin for oomycetes in the mid-Paleozoic, with a divergence between two major lineages, the peronosporaleans and saprolegnialeans, in the early Mesozoic (Figure 1). A complete list of divergence times with 95% confidence intervals for each node under each model is presented in Additional file 2.

**Table 3:** Median divergence times (in Ma) for select nodes estimated under the three molecular clock models

	**Strict clock**		**UCLD relaxed clock**		**Random local clock**	
**Node**^**a**^	**Prior only**	**Full dataset**	**Prior only**	**Full dataset**	**Prior only**	**Full dataset**
a	171.2	26.6	171.5	26.7*	170.6	23.4*
b	271.9	139.9	271.9	119.8*	271.4	134.6
c	167.5	67.0	167.0	71.6*	167.1	75.1
d	363.6	197.2	363.7	191.0*	363.1	214.1
e	83.1	180.1	83.0	97.5*	83.0	139.4
f	183.8	364.4	183.7	191.0	183.8	334.1
g	447.4	414.7	447.4	424.8	447.6	415.6
h	526.6	545.3	527.04	475.0*	527.1	533.9

^a^node as shown in Figure 1. Asterisks (*) indicate ESS values < 200. A complete list of divergence times with 95% confidence intervals is presented in Additional file 2.

**Table 4:** Mean posterior values for select parameters estimated under the three molecular clock models

	**Strict clock**		**UCLD relaxed clock**		**Random local clock**	
**Parameter**	**Prior only**	**Full dataset**	**Prior only**	**Full dataset**	**Prior only**	**Full dataset**
Likelihood	n/a	−359159.81	n/a	−358847.49	n/a	−358852.83
Posterior	n/a	−359328.94	n/a	−358975.87	n/a	−359061.08
Yule.birthrate	0.0052	0.0062	0.0052	0.0074	0.0052	0.0065
Clock.rate	0.9990	0.0018	n/a	n/a	0.9970	0.0019
ucld.mean	n/a	n/a	0.1000	0.0024*	n/a	n/a
ucld.stdev	n/a	n/a	0.0999	0.5550*	n/a	n/a
CoefficientOfVariation	n/a	n/a	0.0979	0.5370*	0.1230	0.2380*
RateChangeCount	n/a	n/a	n/a	n/a	0.6950	8.7050

Asterisks (*) indicate ESS < 200. n/a – not applicable.

## Discussion

Models for estimating divergence times under a molecular clock have become more complex over the past two decades. In this study we have used three distinct models, a single-rate strict clock, a UCLD relaxed clock, and a random local clock, to estimate divergence times among the fungal-like oomycetes. Analyses run under the strict clock model performed robustly, with all parameters showing evidence of thorough sampling (ESS > > 1000) and chain convergence. Because we had no *a priori* expectation of rate homogeneity among oomycetes or between oomycetes and ochrophytes, we also estimated divergence times under “relaxed” clock models. Both the UCLD and random local clock models indicated moderate to high levels of rate variation among lineages (as shown by the coefficient of variation parameter, Table 4), suggesting that a strict clock model was not appropriate for our dataset regardless of performance of the MCMC. In addition, an analysis of Bayes factors suggested that the two relaxed clock methods were a better fit for the data (ln Bayes factor in favor of relaxed clock models over strict clock >100). Rates estimated under the UCLD model appeared to be strongly influenced by the calibration priors, leading to rates 1.5 to 3.5 times higher in the ochrophyte lineages than in the oomycetes (data not shown). However, UCLD analyses failed to converge even after 50 million generations, thus limiting our ability to interpret parameter and divergence time estimates. Only a few parameters showed signs of poor mixing in the random local clock analyses (ESS < 200), but in general there was good evidence of chain convergence under this model, with the trade-off of long computational times.

Despite differences in performance among the three clock models, divergence time estimates among oomycetes were strikingly consistent (Table 3 and Additional file 2), and all models estimated a mid-Paleozoic origin for oomycetes (Figure 1). Our estimate for the divergence of oomycetes from other stramenopiles is somewhat consistent with results from a study of ochrophyte evolution using small subunit ribosomal DNA data [34], but is considerably younger than estimates generated from broader studies of eukaryote evolution [31,32]. However, it seems likely that the times recovered here for the divergence between oomycetes and ochrophytes, as well as the root node, may be underestimated, for several reasons. A recent simulation study of relaxed clock models showed that the deepest nodes in a tree tend to be underestimated when shallow calibrations are used [48], which reflects our reliance on diatom calibrations to estimate divergences throughout the tree. Also, the posterior distributions recovered for the ingroup (node g in Figure 1) and root (node h) time estimates overlapped with their respective prior distributions, and were tightly constrained by the lower limit of 408 Ma imposed by the priors (data not shown). In addition, the long branch connecting the origin of oomycetes (node g) to the divergence between the peronosporaleans and saprolegnialeans (node d), as well as the long branch in the calibration taxa (between nodes e and f), may have influenced rate estimates under the UCLD and random local clock models. As a result, divergence times estimated for these nodes were sensitive to the model, particularly the ochrophyte estimates under the UCLD clock (Table 3 and Additional file 2); however, given the poor performance of the UCLD analysis, it is difficult to assess the reliability of these estimates. Additional sequence data from basal oomycetes such as *Eurychasma dicksonii*[49] and *Haliphthoros* sp. [50], as well as from more ochrophyte calibration taxa, will help break up these long branches and led to more reliable rate estimates. The oldest accepted oomycete fossils come from the Lower Devonian Rhynie Chert, which is thought to have been a non-marine hot spring environment [21,22]. Phylogenetic evidence suggests that the earliest diverging oomycetes were likely marine [15,20], therefore the origin of this group may have occurred some time prior to the appearance of fossils in non-marine environments.

Fossil evidence of oomycetes also occurs throughout the Carboniferous, particularly in association with lycophytes (reviewed in [21]). While previous authors have suggested affinities with certain taxonomic groups (e.g., [25,26]), the divergence times estimated here indicate that modern peronosporalean and saprolegnialean lineages originated much later, in the mid to late Mesozoic (Figure 1). Modern saprolegnialeans, such as *Saprolegnia parasitica*, are commonly associated with freshwater environments, and can be devastating pathogens of fish, amphibians, crustaceans, and insects [45]; saprotrophic species, such as *Thraustotheca clavata*, are also known from this group. In contrast, modern peronosporaleans are predominately terrestrial and many are significant plant pathogens. Two species included in our analysis, *Hyaloperonospora arabidopsidis*[39] and *Albugo laibachii*[37], are obligate biotrophs who are fully dependent on their host (*Arabidopsis*). *Phytophthora* species cause disease on a wide variety of plants, and significant effort has been undertaken to understand their mechanisms of virulence and host specificity (reviewed in [51]). While it is undesirable to extrapolate as to the likely hosts for early diverging lineages, it does seem reasonable to suggest that host availability was not a constraining factor in oomycete diversification. Particularly for the modern plant pathogenic oomycetes, both fossil and molecular clock evidence suggests that the major lineages of angiosperms had diversified by the mid-Cretaceous [52], prior to our estimates for divergences among the peronosporaleans. The evolution of pathogenic lifestyles, therefore, may have been in response to certain environmental changes, or may have been facilitated by the horizontal transfer of pathogenicity-related genes from true Fungi [53-55] or from bacteria [45,56], as has been suggested previously.

In this study, we chose to focus on conserved regulators of eukaryotic gene expression to examine their presence and level of conservation in pathogenic oomycetes. Mechanisms of gene expression regulation are highly conserved across eukaryotes and were most likely present in the last common ancestor, including epigenetic and RNA-based processes for transcriptional and post-transcriptional gene silencing [57-59]. Although we have not conducted an exhaustive survey here, our results suggest that the common ancestor of oomycetes possessed a full complement of regulatory proteins, including those involved in histone modification, RNA interference, and tRNA and rRNA methylation. Surprisingly, no orthologs of canonical DNA methyltransferases could be identified in the genomes of oomycetes. A single putative DNA methylase is present in the genome of *Pythium ultimum* (T014901), but no orthologs could be detected in the other oomycete genomes. Gene silencing studies in *Phytophthora infestans* have failed to detect evidence of cytosine methylation [60,61], however recent work in *P. sojae* does suggest the presence of methylated DNA [62]. DNA methyltransferases also appear to be absent from the *Ectocarpus* genome [38], as well as from the model eukaryotes *Saccharomyces cerevisiae* and *Caenorhabditis elegans*[63], however several are known from diatoms [64,65]. Further study is therefore needed to confirm the presence and mechanism of DNA methylation in oomycetes.

## Conclusions

This is the first study to estimate divergence times among the fungal-like oomycetes. The consistency of our time estimates under three distinct molecular clock models suggests that the resulting timetree likely recovers the main divergences among lineages, which occurred in the mid to late Mesozoic. Our estimates for the origin of oomycetes and the divergence of stramenopiles from other eukaryotes may have been underestimated due to the limited fossil information available for the taxa included in this study. Additional information from the oomycete fossil record, especially from the diverse Cretaceous assemblages, as well as new sequence data from basal oomycete lineages and other under-sampled eukaryotes [66], may help future molecular clock studies better estimate evolutionary rates.

## Methods

### Data mining

Reference sequences for canonical eukaryotic transcription factors and proteins involved in post-transcriptional gene silencing, DNA and RNA methylation, and chromatin modification were obtained from the Gene Database at NCBI (http://www.ncbi.nlm.nih.gov/gene) for human, *Drosophila*, *Saccharomyces*, and/or *Arabidopsis*. The reference protein sequences were then used to search for homologs in the genome of *Phytophthora infestans* T30-4 [42]. Additional reference sequences were also obtained from a study of gene silencing in *P. infestans*[36]. Both the eukaryotic reference sequences and the putative *P. infestans* homologs were used to search the available genomes of oomycetes, diatoms, and a brown alga (Table 1); outgroup sequences were obtained from *Tetrahymena thermophila* when available. All potential homologs of equivalent BLAST e-values within each genome were included for orthology assessment.

### Dataset assembly

Protein domains were determined for all potential homologs using Pfam [67]. Sequences that did not contain the appropriate domains for proper protein function were removed from each dataset except in cases where the protein sequence appeared truncated due to genome misannotation, particularly for *Hyaloperonospora arabidopsidis*. Each dataset was aligned under default settings in ClustalX v2 [68], and preliminary neighbor-joining phylogenies were generated under a Poisson correction with pairwise deletion of alignment gaps in MEGA v5 [69]. Sequences within each dataset were considered orthologous if they shared protein domains and their phylogeny reflected known species relationships. In datasets with species-specific paralogs, one sequence was arbitrarily chosen to represent the ortholog for divergence time estimation. In cases where orthology was ambiguous or no homolog could be identified, the sequence was coded as missing data. A complete list of protein accession numbers per gene for each genome is available in Additional file 1.

### Divergence time analysis

Protein datasets with robust orthology were used to co-estimate phylogeny and divergence times using Bayesian inference in BEAST v1.7.5 [70]. Initial runs of 10 million generations were used under each clock model to evaluate settings on priors and to generate a user tree for subsequent analyses. For the final analyses, each protein dataset was treated as a separate partition under a WAG substitution model; a Yule speciation process was assumed with a uniform distribution on the birthrate (0–100; initial value 0.01). For the strict clock analyses, the rate parameter (*clock.rate*) was modeled with an exponential prior distribution (mean 1.0, initial value 0.01). For the UCLD relaxed clock model, an exponential prior distribution (mean 0.1, initial value 0.01) was used for the mean rate (*ucld.mean*) and standard deviation (*ucld.stdev*). Several parameters control the rate and number of rate changes under the random local clock model; a Poisson distribution (mean 0.693) was used as the prior for the number of local clocks (*rateChanges*), an exponential prior distribution (mean 1.0, initial value 0.001) was used for the relative rates among local clocks (*localclocks.relativerates*), and an exponential prior distribution (mean 1.0, initital value 0.01) was used for the rate (*clock.rate*). Five independent analyses were run for 50 million generations each, under all three clock models; log and tree files from the two runs with the highest parameter ESS values per model were combined (after removing burn-in from each run) using LogCombiner v1.7.5. Tracer v1.5 [71] was used to evaluate convergence, estimate the appropriate burn-in for each run, and calculate Bayes factors for model comparisons. Analyses were also repeated without data (priors only) to determine the impact of calibration settings on the resulting divergence time estimates; three independent runs of 50 million generations each were performed under each clock model. Trees were visualized in FigTree v1.4 [72].

Fossil evidence from diatoms and oomycetes was used to calibrate the molecular clock analyses; all calibrations were modeled with a gamma prior distribution (shape 2.0) with the offset value set as the uppermost boundary of the time interval (stage) containing the relevant fossil. The value for the scale parameter was set so that the age at the 95% quantile was roughly equivalent to the lowermost boundary on the geologic epoch containing the relevant fossil. Appropriate geological times were obtained from the International Commission on Stratigraphy chronostratigraphic chart, January 2013 version (http://stratigraphy.org). Fossil evidence from the Late Cretaceous (Campanian) pennate diatoms [73] provided a minimum age of 72.1 Ma on the divergence between *Thalassiosira* and *Phaeodactylum* (5-95% quantiles = 74–100 Ma). Early Jurassic (Toarcian) diatom fossils [74] provided a minimum age of 174 Ma on the divergence between diatoms and *Ectocarpus* (5-95% quantiles = 176–202 Ma). The Rhynie chert oomycete fossils [21] were used to define a minimum divergence time of 408 Ma between oomycetes and ochrophytes (5-95% quantiles = 418–550 Ma). A wide uniform prior distribution (408–1750 Ma; initial value 635 Ma) was used for the root age as there are few robust estimates on the divergence between alveolates and stramenopiles. Beast XML-formatted data files have been deposited in Dryad [75].

## Availability of supporting data

The data sets supporting the results of this article are available in the Dryad Digital Repository, http://dx.doi.org/10.5061/dryad.39mc5.

## Competing interests

The authors declare that they have no competing interests.

## Authors’ contributions

JEB and NHM designed the experiment and performed the data mining. JEB performed the divergence time analyses and drafted the manuscript. Both authors read and approved the final manuscript.

## Supplementary Material

Additional file 1 Genome accession numbers for all proteins included in this study. Click here for file

Additional file 2 Timetree, divergence times, and 95% confidence intervals per node estimated under the three clock models. Click here for file
