# Supplementary material for: A multilocus timescale for oomycete evolution estimated under three distinct molecular clock models
Source: BMC Evol Biol. 2014 May 12;14:101. doi: 10.1186/1471-2148-14-101 (PMC4030286; doi:10.1186/1471-2148-14-101)
Supplement: Additional file 2 — Timetree, divergence times, and 95% confidence intervals per node estimated under the three clock models. [file 1471-2148-14-101-S2.pdf]

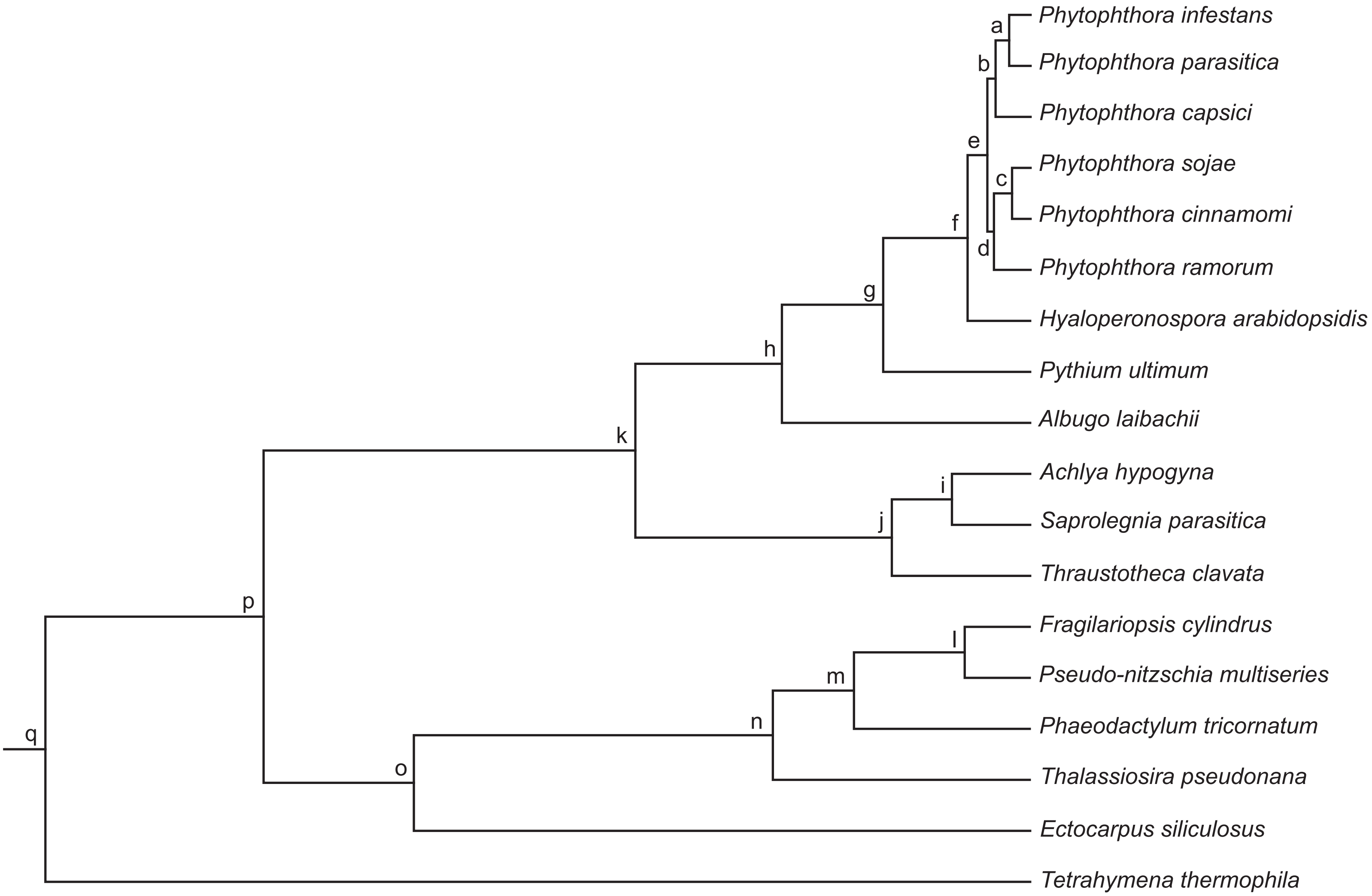

	Strict Clock		UCLD Clock		Random Local Clock	
Node	Divergence	95% CI	Divergence	95% CI	Divergence	95% CI
a	13.0	(12.1, 13.9)	11.6	(5.1, 20.0)	11.6	(8.2, 13.8)
b	21.5	(20.4, 22.6)	20.4	(10.6, 31.4)	18.9	(15.8, 21.4)
c	10.9	(10.1, 11.8)	10.0	(4.7, 17.1)	10.1	(8.2, 11.6)
d	22.3	(21.3, 23.5)	21.1	(12.3, 32.6)	19.8	(16.3, 22.2)
e	26.6	(25.5, 27.9)	26.7	(17.3, 39.0)	23.4	(19.8, 26.2)
f	39.9	(38.1, 41.8)	41.9	(25.6, 61.3)	34.0	(29.1, 37.7)
g	86.3	(83.2, 89.9)	67.8	(39.2, 115.2)	79.8	(74.8, 84.9)
h	139.9	(135.6, 145.3)	119.8	(71.7, 186.8)	134.6	(127.5, 142.4)
i	41.4	(39.5, 43.6)	40.7	(17.2, 68.3)	42.4	(39.3, 45.7)
j	67.0	(64.4, 70.0)	71.6	(36.9, 117.3)	75.1	(71.1, 79.2)
k	197.2	(191.6, 204.2)	191.0	(125.2, 269.6)	214.1	(205.3, 224.2)
l	55.2	(52.8, 57.7)	30.7	(18.7, 44.7)	35.3	(31.5, 38.2)
m	134.3	(130.0, 139.6)	70.3	(51.2, 89.2)	95.3	(85.4, 102.3)
n	180.1	(174.6, 186.9)	97.5	(77.1, 118.7)	139.4	(132.7, 146.9)
o	364.4	(355.0, 377.1)	191.0	(174.3, 221.1)	334.1	(322.9, 348.2)
p	414.7	(408.1, 427.1)	424.8	(408.3, 457.5)	415.6	(408.2, 429.7)
q	545.3	(530.3, 564.8)	475.0	(412.1, 598.8)	533.9	(516.6, 556.1)
